# The Effects of CPAP Treatment on Resting-State Network Centrality in Obstructive Sleep Apnea Patients

**DOI:** 10.3389/fneur.2022.801121

**Published:** 2022-03-28

**Authors:** Panmei Li, Yongqiang Shu, Xiang Liu, Linghong Kong, Kunyao Li, Wei Xie, Yaping Zeng, Haijun Li, Dechang Peng

**Affiliations:** ^1^Medical Imaging Center, The First Affiliated Hospital of Nanchang University, Nanchang, China; ^2^Positron Emission Tomography (PET) Center, The First Affiliated Hospital of Nanchang University, Nanchang, China

**Keywords:** obstructive sleep apnea, continuous positive airway pressure, resting-state fMRI, degree centrality, cognitive impairment

## Abstract

**Background and Objectives:**

Obstructive sleep apnea (OSA) is the most common sleep disorder and previous studies have shown that OSA patients suffer from brain network impairments associated with cognitive deficits, and continuous positive airway pressure (CPAP) treatment can improve clinical symptoms. However, the relationship between CPAP treatment and brain network changes remains unclear. This study explored the characteristics of brain network changes in OSA patients before (pre-CPAP) and after one month of CPAP treatment (post-CPAP).

**Methods:**

We collected data, including sleep monitoring, clinical assessment, and magnetic resonance imaging scans, from 21 OSA patients and 21 age-matched healthy controls (HCs). Voxel-level degree centrality (DC) was used to assess whole-brain network connectivity characteristics, a two-sample *t*-test was used to compare network differences between pre-CPAP OSA patients and HCs, and a paired sample *t*-test was used to compare the characteristics of brain network changes in OSA patients before and after treatment. The correlations between the DC value and each of the clinical variables were analyzed in the OSA patients.

**Results:**

Compared with HCs, pre-CPAP OSA patients showed increased DC values in the bilateral cerebellar posterior lobes (CPLs) and decreased DC values in the right superior temporal gyrus, left superior frontal gyrus and right middle frontal gyrus. Compared with pre-CPAP OSA patients, post-CPAP OSA patients showed reduced DC values in the bilateral CPL and increased DC values in several brain regions in the frontal, temporal, and insular lobes after CPAP treatment. The Montreal Cognitive Assessment MoCA (MoCA) scores were positively correlated with the DC value of the bilateral cerebellum posterior lobe, right middle temporal gyrus, left superior temporal gyrus, left paracentral lobule and left paracentral lobule. Also, Pittsburgh Sleep Quality Index (PSQI) scores were negatively correlated with the DC value of the right middle temporal gyrus in post-CPAP OSA patients.

**Conclusion:**

CPAP treatment can effectively reverse the compensatory response of the bilateral CPL and functional network damage brought about by OSA, which may provide potential neuroimaging biomarkers for CPAP treatment evaluation.

## Introduction

Obstructive sleep apnea (OSA), characterized by recurrent apnea and hypopnea events during sleep and accompanied by intermittent hypoxemia, sleep fragmentation, and daytime sleepiness (1, 2), is the most common sleep disorder (3, 4). An epidemiological review reported that ~13–33% of adult males and 6–19% of adult females suffer from OSA (4, 5). OSA patients are prone to show multiple psychological comorbidities, including cognitive deficits and mood symptoms (6–8); however, the exact pathophysiological mechanisms of underlying psychological comorbidities in OSA patients remain unclear. Previous studies found multiple cognitive-related brain regions and brain network abnormalities in OSA patients by exploring brain function, brain structure, cerebral metabolism and regional cerebral blood flow (9–14); these abnormalities were mainly located in the cerebellum, prefrontal, temporal area and hippocampus.

Continuous positive airway pressure (CPAP) is considered to be the most effective and widely used method to prevent upper airway collapse in adults with OSA (15, 16), improve hypoventilation and apnea events in OSA patients during sleep and slow the disease progression (17). Although the effectiveness of CPAP treatment to improve cardiovascular response, inflammation and especially cognitive performance was generally recognized, there was inconsistent recovery of neurological symptoms in patients (18–20), suggesting differential effect on brain function behind these processes. However, the underlying neural mechanisms of differential remodeling of cognitive impairment after CPAP treatment in OSA patients are poorly understood.

Neuroimaging methods broadened new ideas for the study of neural mechanisms. Morphometric studies demonstrate that CPAP treatment can not only redress cognitive but also neurostructural deficits (21, 22), which have provided evidence that brain structures can recover following treatment. However, the region of brain volume recovery have not been consistently observed in all studies (23). A white matter (WM) integrity and metabolism study demonstrated that CPAP therapy activates the brain's healing mechanisms by reversing hypoxia, thereby improving the microstructural integrity and perfusion of the brain and reducing the damage in the brain (24). Jensen found that CPAP treatment could normalize reduced cerebral blood flow in OSA patients exposured to hypoxia, but there was no change in cerebral oxygen metabolism and cerebral lactate (25). A previous study showed that CPAP treatment reduces cognitive deficits in older patients and suggests that CPAP may be effective in improving brain function by increasing default mode network (DMN) connectivity and reducing cortical thinning (26), however, age-related functional decline may affect the generalization of the findings. The above studies suggested that cognitive impairment and brain neurological damage induced by OSA are reversible. However, previous studies have focused on the effects of brain structure, blood flow and metabolism in OSA patients, with very few studies on resting-state brain networks, and the regression of brain networks in OSA patients after treatment remains unclear.

The human brain is a highly complex network system consisting of interactions of neurons and neuronal clusters (27). Voxelwise degree centrality (DC) explores the properties of the whole-brain functional connectome at the voxel level and is the most direct analysis method to describe the influence and function of nodes (28). It does not require a priori assumptions and can directly assess the connectivity patterns of whole brain functional networks, thus tapping into the physiological basis of the brain's intrinsic functional framework. Unlike independent component analysis or seed-based functional connectivity, voxel-based DC measurement directly and quantitatively reflects changes in the functional relationships of a given voxel or node across functional brain network connectomes. Our previous study found that OSA patients revealed specific intrinsic abnormalities in the topological characteristics of functional networks in OSA, including reduced DC values in the prefrontal, occipital, cingulate, and parietal lobes and increased DC values in the orbital frontal cortex, cerebellum posterior lobes, and lentiform nucleus. These findings suggested that OSA patients have abnormalities in functional network integration and segregation and impairment and compensatory changes in functional hub nodes (11). Previous studies have also confirmed the high sensitivity, specificity, and reproducibility of DCs (29), and it has been increasingly applied to study neural network damage in diseases associated with cognitive impairment and to assess the plasticity of brain damage after treatment, such as Parkinson's disease, glaucoma, and depression (30–33). However, the pattern of changes in the global brain network in OSA patients after CPAP therapy is unclear.

Based on the above questions, we proposed the scientific hypothesis that CPAP treatment could partially improve functional brain network impairment and associated cognitive function in OSA patients. To address this question, we used a voxel-based DC approach to first compare the differences in brain networks between pre-CPAP OSA patients and HCs. Then, we compared the characteristics of the changes in OSA patients' own brain networks before and after CPAP treatment; finally, we explored the relationship between the changes in brain functional networks and clinical assessment.

## Materials and Methods

### Subjects

All patients diagnosed with obstructive sleep apnea who were ready for CPAP treatment at the Sleep Monitoring Room at the Respiratory Department of the First Affiliated Hospital of Nanchang University were considered for inclusion in this study. None of the patients had been treated by CPAP before the study. The diagnosis of patients was made jointly by 2 experienced respiratory physicians based on criteria from the American Academy of Sleep Medicine (AASM) 2017 proposed clinical practice guidelines for obstructive sleep apnea in adults (34). Patients with OSA, indicated by an apnea hypopnea index (AHI) >15/h, were selected for this study. All participants were right-handed, native Chinese speakers, aged from 22 to 60. The exclusion criteria for all subjects were as follows: [1] sleep disorders other than OSA; [2] respiratory disease; cardiovascular disease; diabetes; hypothyroidism; or history of central nervous system disease; [3] alcohol or illicit drug abuse or current psychoactive drug use; and [4] contraindications to MRI. The same exclusion criteria were also applied to healthy controls (age- and education-matched) recruited from the community and excluding those with polysomnography (PSG) monitoring AHI >5 times/h or a history of sleep disorders.

This was a longitudinal study; for each OSA patient, we obtained brain MRI data and performed a neuropsychological assessment at baseline and one month after CPAP treatment follow-up. Healthy controls were assessed at baseline only. The study was approved by the Medical Research Ethics Committee of The First Affiliated Hospital of Nanchang University [No. 2020(94)]. All subjects volunteered to participate in the experiment and signed written informed consent.

### Polysomnography and Neuropsychological Assessments

All subjects were required to undergo overnight PSG and were required to avoid hypnotics, alcohol and coffee for one day before PSG. A PSG monitor (Alice 5 LE, Respironics, Orlando, FL, USA) was used to record items, including standard electroencephalogram (EEG), electrooculogram (EOG), chin electromyogram (EMG), electrocardiogram (ECG), body position, thoracic and abdominal respiratory movements, oral and nasal airflow, and snoring. Total sleep time, sleep efficiency, sleep latency, oxygen saturation (SaO2), sleep stages, arousal, and respiratory events were likewise recorded (34), obstructive apnea was defined as a reduction in airflow ≥90% or a continuous absence of airflow for at least ≥10 s. Hypopnea was defined as a reduction in airflow ≥30% with 4% or higher oxygen desaturation accompanied by ≥4% SaO2. The apnea hypopnea index (AHI) was obtained from the aggregate numbers of apnea and hypopnea events per hour of patients' sleep.

All participants completed neuropsychological assessments, including the Epworth Sleepiness Scale (ESS), PSQI, MoCA, Hamilton Anxiety Scale (HAMA), and Hamilton Depression Scale (HAMD), under the guidance of a professional neuropsychologist in a quiet state. Daytime sleepiness and subjective sleep quality were assessed by ESS and PSQI, respectively. ESS scores ranged from 0 to 24 (8 different categories from 0 to 3), with scores above 6, 11, 16 representing sleepy, excessive sleepiness, and excessive sleepiness, respectively. The total score of PSQI is 0–21,and the higher score means the worse sleep quality. Cognitive function was assessed using the 11-item MoCA, examined in 8 cognitive domains, including executive function, language, attention, computation, abstraction, naming, memory, and orientation. A total MoCA score of <26 indicated cognitive impairment. Patients also completed a questionnaire for the assessment of psychiatric symptoms, using the 17-item HAMD rating scale and the HAMA scale to assess depressive symptoms and anxiety symptoms, respectively. A total HAMD score over 24 may have indicated severe depression; a total score over 17 may have indicated mild to moderate depression; and a total score <7 may have indicated no depressive symptoms. A total HAMA score over 29 may be severe anxiety; over 21 must have significant anxiety; over 7 may have anxiety; if <7 has no anxiety symptoms.

### CPAP Treatment

All patients proposed for CPAP treatment after clinician health education intervention were treated with a standardized auto adjustment model of CPAP (YH-480, Yuwell, Jiangsu, China). Patients underwent automatic pressure titration on the night of sleep using a ventilator with a therapeutic pressure setting of 4–20 cm H_2_O and c. The duration of treatment is at least 1 month, and the frequency is at least 5 days per week, at least 4 h per night. The duration of machine use is automatically recorded by the ventilator's built-in IC card, giving us evidence of compliance.

### MRI Data Acquisition

All participants collected MRI images in a 3.0 Tesla MRI scanner of an 8-channel phased-array head coil (Siemens, Erlangen, Germany) in our hospital. The acquisition was first performed the day after PSG monitoring (between 7 and 9 p.m.), and then after completion of at least one month of qualifying CPAP therapy. Foam pads and ear plugs were used to reduce the patient's head movements and to minimize the noise of the scanner. Before the scan, all the subjects need to close their eyes, be awake, and lie quietly on the examination bed without performing specific thinking activities. First, a routine MRI scan of the skull was performed with the following parameters: conventional T1-weighted imaging [repetition time (TR) = 250 ms, echo time (TE) = 2.46 ms, thickness = 5 mm, gap = 1.5 mm, field-of-view (FOV) = 220 × 220 mm, slices = 19] and T2-weighted imaging (*TR* = 4,000 ms, TE = 113 ms, thickness = 5 mm, gap = 1.5 mm, FOV = 220 × 220 mm, slices = 19). Then, high-resolution T1-weighted brain structural MRI images were obtained from each subject using a brain volume sequence in the sagittal plane (TR = 1,900 ms, TE = 2.26 ms, thickness = 1.0 mm, gap = 0.5 mm, FOV = 250 × 250 mm, matrix = 256 × 256, flip angle = 9°, slices = 176). Finally, Rs-fMRI data were acquired with a gradient-recalled echo planar imaging (EPI) sequence in the axial plane (TR = 2,000 ms, TE = 30 ms, flip angle = 90°, thickness = 4.0 mm, gap = 1.2 mm, FOV = 230 × 230 mm^2^, matrix size = 64 × 64, slices = 30), and 240 rs-fMRI images in total were recorded. Two experienced radiologists read the images to rule out gross brain parenchymal lesions such as brain infarcts and tumors, and no subjects were excluded.

### Data Preprocessing

The imaging data were examined by MRIcro software (www.MRIcro.com) to discard suboptimal data. Data were preprocessing by the Data Processing & Analysis Assistant for Resting-State Brain Imaging (DPABI, Chinese Academy of Sciences, Beijing, China, http://rfmri.org/dpabi), which was based on statistical parametric mapping (SPM12, http://www.fil.ion.ucl.ac.uk/spm/software/spm12/) and was running on MATLAB2018b (Math Works, Natick, MA, USA). First, the file format was converted from DICOM to NIFTI. Then, the remaining time series were processed for slice timing and three-dimensional head motion correction. Participants with a maximum cardinal direction displacement (x, y, z) > 2.0 mm, maximum spin (x, y, z) > 2.0° or framewise displacement (FD) of any of the 230 volumes exceeding 2.5 standard deviations were excluded (35). The structural images were co-registered to the functional images of each subject using a linear transformation. Consequently, the new segmentation in SPM8 was used to segment all subjects' structural images into white matter and cerebrospinal fluid (CSF). Then, the images were spatially normalized to the Montreal Neurological Institute (MNI) template and resampled to 3 × 3 × 3 mm voxels. Finally, linear regression was used to regress out the Friston 24 parameter, white matter signal, and cerebrospinal fluid signal from the time series of all voxels, and the resulting images were smoothed using a Gaussian kernel of 6-mm full width at half-maximum after filtering with a temporal filter (0.01–0.08 Hz).

### Voxelwise Degree Centrality

REST (http://www.restfmri.net) software was used to extract DPABI default whole brain gray matter template (61 × 73 × 61, 3 × 3 × 3 mm, 67,541 voxels). We calculated Pearson's correlation, in the default gray matter template, between any pair of voxels for each subject. We calculated Voxelwise DC on the basis of the following formula (28):


(1) $$\begin{array}{r} DC\left(i\right)\;=\;\overset{N}{\underset{j\;=\;1}{\sum }}r_{ij}\left(r_{ij}\;>\;r_{0}\right) \end{array}$$

The correlation coefficient between voxel i and voxel j is represented as rij, and the correlation threshold sat to eliminate weak correlations is called r_0_ (36). Then, we applied five different correlation thresholds (r_0_ = 0.15, 0.2, 0.25, 0.3 and 0.35) in this study to increase the stability and repeatability (30). We converted the correlation coefficient into a Z-score map using Fisher transformation to improve normality, and then smoothed the z-score map with a 6 mm Gaussian kernel of full width at half maximum.

### Statistical Analysis

In terms of demographic and clinical data, we first used SPSS 25.0 software (IBM SPSS 25.0, Chicago, IL, USA) to verify the data distribution by the Kolmogorov–Smirnov test. Then, two samples *t*-tests and Mann–Whitney U-tests were applied to normally distributed continuous data and non-normally distributed data, respectively. Gender proportions were tested by χ^2^ test. *p* < 0.05 indicates that the difference is statistically significant. Paired-sample *t*-test was used to analyze the data difference between the post- and pre-CPAP OSA patients. *P* values were corrected using Bonferroni correction at a level of *P* < 0.017 (0.05/3).

For the voxel-based DC analysis, first, a one-sample *t*-test was performed to identify the spatial pattern of intrinsic functional hubs in the two groups in the DC maps. Second, we compared the differences in DC values between OSA patients and HCs at the baseline level using an independent sample *t*-test with the REST V1.8 software package. Third, a paired sample *t-*test was used to compare the differences in DC values between OSA patients before and after CPAP treatment. All results were reported at the significant voxel level of *P* < 0.01, cluster > 40 voxels, AlphaSim corrected. Finally, to assess the relationship between clinical variables and DC values with significant differences before and after treatment, we applied Pearson correlation or Spearman correlation analysis to evaluate data with continuous normal distribution and data with non-normal distribution, respectively. *P* < 0.05 was deemed to be statistically significant.

## Results

### Demographic and Clinical Characteristics

The demographic and clinical characteristics of OSA patients and HCs are summarized in Table 1. We found no significant divergences in sex, age, or education years between the pre-CPAP OSA patients and HCs (*P* > 0.05). Compared with HCs, patients in the pre-CPAP OSA group had meaningful higher scores for BMI, AHI, SaO2 <90%, ESS, PQSI, HAMA, HAMD, and MoCA and had significantly lower scores for sleep efficiency, nadir SaO2 and mean SaO2. After one month of standard CPAP treatment, we found that the OSA patients showed significantly decreased ESS, HAMA and HAMD scores and increased MOCA scores after CPAP compared with those before treatment. However, PSQI as well as MOCA differences were not further corrected by Bonferroni correction.

**Table 1:** Population and clinical characteristics of participants.

**Characteristics**	**HC groups (*N* = 21)**	**pre-CPAP OSA (*N* = 21)**	**post-CPAP OSA (*N* = 21)**	***P* value (pre-CPAP OSA vs. HC)**	***P* value (post-CPAP vs. pre-CPAP OSA)**
Sex (male/female)	20/1	20/1	20/1	1.000	/
Age (year)	40.1 ± 8.6	40.1 ± 8.4	/	1.000	/
Snoring History (year)	10.2 ± 6.3	/	/	/	/
Education (years)	10.1 ± 3.6	12.4 ± 3.8	/	0.033	/
AHI	2.5 ± 1.9	48.4 ± 19.5	2.8 ± 2.21	<0.001	<0.001
Nadir SO_2_ (%)	94.0 ± 3.27	69.8 ± 13.6	/	<0.001	/
mean SO_2_ (%)	97.3 ± 1.9	93.7 ± 3.3	/	<0.001	/
SaO2 <90 (%)	0.3 ± 0.2	15.0 ± 17.4	/	<0.001	/
Sleep efficiency (%)	90.5 ± 3.1	74.6 ± 24.0	/	0.007	/
BMI (kg/m^2^)	21.1 ± 1.5	27.3 ± 3.8	27.2 ± 4.0	<0.001	0.770
ESS	2.8 ± 1.9	10.9 ± 5.3	8.0 ± 4.2	<0.001	0.007
HAMA	4.4 ± 2.3	7.5 ± 4.6	4.0 ± 3.7	<0.001	<0.001
HAMD	3.1 ± 1.5	6.2 ± 3.1	3.0 ± 1.7	<0.001	<0.001
PSQI	1.6 ± 1.1	5.5 ± 1.9	4.1 ± 2.2	<0.001	0.020
MoCA	27.5 ± 1.5	23.1 ± 3.0	24.5 ± 3.8	<0.001	0.040
mean CPAP use (h/night)	/	/	5.6 ± 1.3	/	/
days of CPAP use > 4 h(%)	/	/	65.7 ± 19.8	/	/
mean CPAP pressure (cmH2O)	/	/	9.2 ± 1.7	/	/

*HC, healthy controls; pre-CPAP OSA, OSA patients before CPAP treatment; post-CPAP OSA, OSA patients after CPAP treatment; BMI, body mass index; AHI, apnea hypopnea index; SaO2, oxygen saturation; SaO2 <90%, percentage of total sleep time spent at oxygen saturation <90%; ESS, Epworth sleepiness scale; HAMA, Hamilton Anxiety Scale; HAMD, Hamilton Depression Scale; PSQI, Pittsburgh Sleep Quality Index; MoCA, Montreal Cognitive Assessment*.

### Degree Centrality Difference in Pre-CPAP OSA and HC

**Figure 1:**
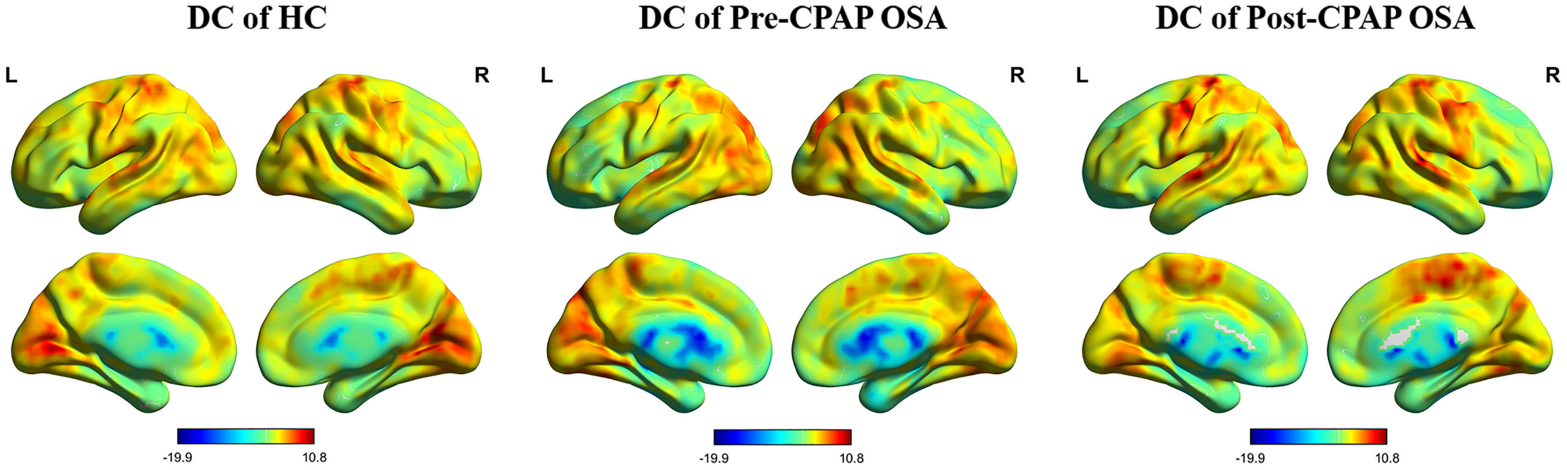
Highly similar spatial distributions of the higher DC value in patients in the HC group, pre-CPAP OSA group and post-CPAP OSA group (r_0_ = 0.25).

**Figure 2:**
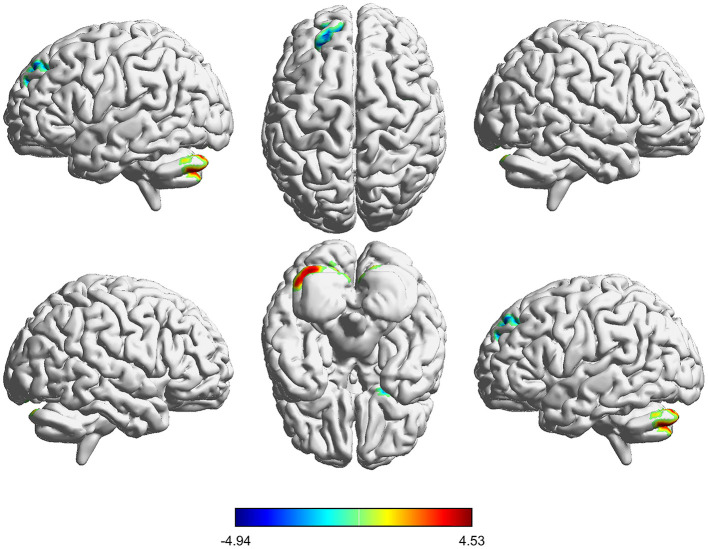
Voxelwise comparison of DC between participants in the pre-CPAP and HC groups. Brain clusters showing a significant difference in degree centrality (DC) in the pre-CPAP OSA patients compared with those of participants in the HC group. Hot color (cool) indicates significantly increased (decreased) DC in the pre-CPAP patients.

Compared to the global mean value, both groups displayed similarly higher DC value (functional hub) spatial distributions in the prefrontal, cingulate and temporal cortical regions (Figure 1). The intergroup differences were remarkably similar at different correlation thresholds (r_0_ = 0.15, 0.2, 0.25, 0.3 and 0.35), which proved the stability of the result (Supplementary Figure 1). Therefore, we primarily reported the results based on the weighted graph of positive correlations (r_0_ = 0.25). Compared with HCs, OSA patients showed significantly increased DC in the bilateral cerebellum posterior lobe (CPL) but significantly lower DC values in the right superior temporal gyrus (STG), left superior frontal gyrus (SFG) and right middle frontal gyrus (MFG) (Table 2; Figure 2).

**Table 2:** Brain areas showed significant differences in DC between pre-CPAP OSA patients and HCs (r_0_ = 0.25).

**Condition**	**Brain region**	**L/R**	**MNI coordinates**			**Num. of voxels**	**T values**
			**x**	**y**	**z**		
OSA>HC	CPL	L	−33	−81	−36	294	4.534
	CPL	R	15	−87	−27	46	4.275
OSA < HC	STG	R	37	9	−27	51	−4.873
	SFG	L	−21	45	42	80	−4.593
	MFG	R	27	3	45	42	−4.938

*DC, degree centrality; pre-CPAP OSA, OSA patients before CPAP treatment; HC, healthy control; CPL, cerebellum posterior lobe; STG, superior temporal gyrus; SFG, superior frontal gyrus; MFG, middle frontal gyrus; L, left; R, right*.

### Degree Centrality Difference in OSA Patients Before and After CPAP

**Figure 3:**
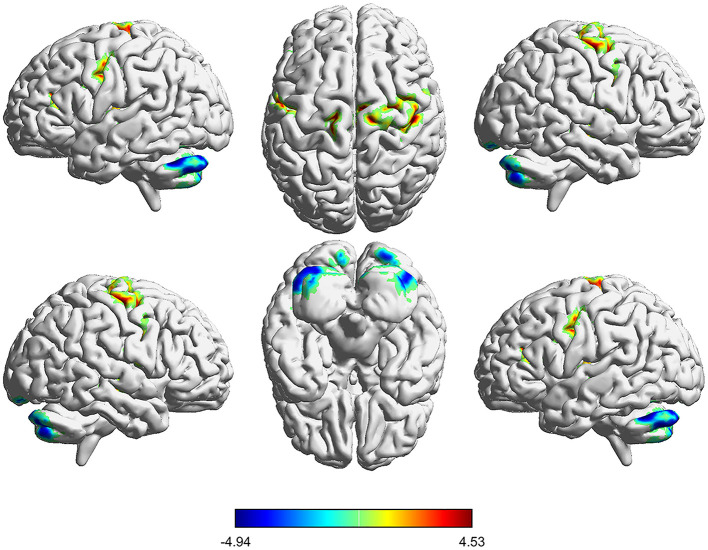
Voxelwise comparison of DC between pre-CPAP OSA and post-CPAP OSA. Brain clusters showing a significant difference in degree centrality (DC) between OSA patients before and after CPAP treatment (cluster-level p <0.01, GRF correction). Hot color (cool) indicates significantly increased (decreased) DC in the post-CPAP patients.

**Figure 4:**
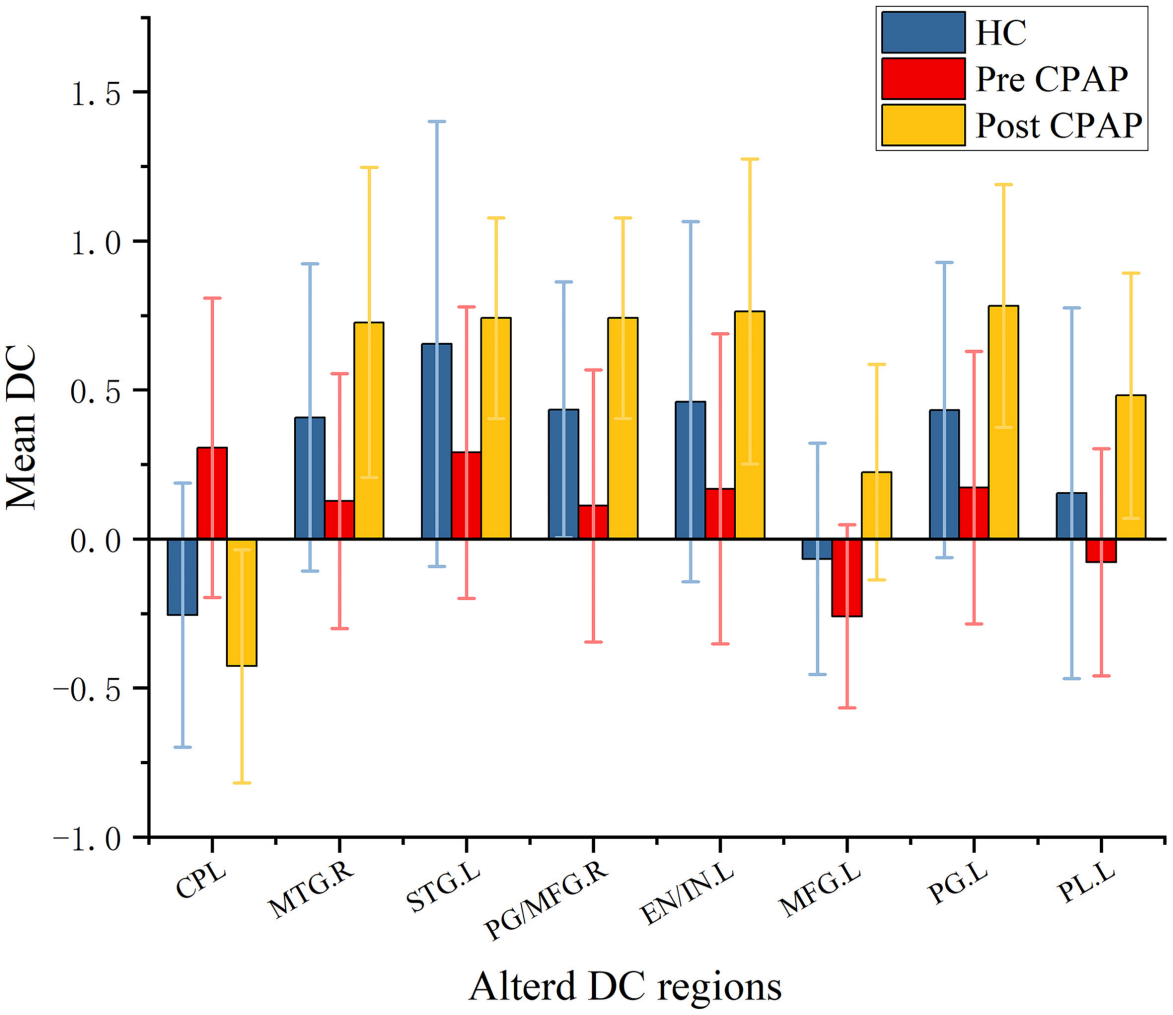
Mean weighted DC signal values of HCs and pre- and post-CPAP OSA patients in altered regional brain areas before and after CPAP treatment. DC, Degree Centrality; pre-CPAP OSA, OSA patients before CPAP treatment; post-CPAP, OSA patients after CPAP treatment; HC, Healthy controls; CPL, Cerebellum posterior lobe; MTG, Middle temporal gyrus; STG, Superior temporal gyrus; PG, Precentral gyrus; MFG, Middle frontal gyrus; PL, Paracentral lobule; L, Left; R, Right.

These results were also remarkably similar at different correlation thresholds (r_0_ = 0.15, 0.2, 0.3 and 0.35) between post- and pre-CPAP OSA patients (Supplementary Figure 2). Compared with the pre-CPAP OSA measurements, the post-CPAP OSA measurements showed significantly decreased DC in the bilateral PCL, and there was higher DC between patients in the pre-CPAP and HC groups. DC values increased in several brain regions, including the right middle temporal gyrus (MTG), left STG, bilateral precentral gyrus (PG) and MFG, left extranuclear and insula, left MFG, and left paracentral lobule (PL) (Table 3; Figure 3). Interestingly, either the decreased or increased DC values of all the above regions were lower or higher than the DC values of the corresponding brain regions in the HC groups (Figure 4).

**Table 3:** Brain areas showed significant differences in DC between pre-CPAP OSA and post-CPAP OSA patients (r_0_ = 0.25).

**Condition**	**Brain region**	**L/R**	**MNI coordinates**			**Num. of voxels**	**T values**
			**x**	**y**	**z**		
post-CPAP < pre-CPAP OSA	CPL	L/R	−3	−90	−27	1,105	−7.592
post-CPAP > pre-CPAP OSA	MTG	R	54	−18	−12	47	4.064
	STG	L	−45	−18	0	71	4.525
	PG/MFG	R	9	−21	57	754	7.205
	EN/Insula	L	−33	−6	−9	105	5.033
	MFG	L	−36	33	18	51	4.172
	PG	L	−54	−6	42	85	4.177
	PL	L	−12	−12	60	107	5.813

*DC, based voxel degree centrality; pre-CPAP OSA, OSA patients before CPAP treatment; post-CPAP OSA, OSA patients after CPAP treatment; HC, healthy controls; CPL, cerebellum posterior lobe; MTG, middle temporal gyrus; STG, superior temporal gyrus; PG, precentral gyrus; EN, extranuclear; MFG, middle frontal gyrus; PL, paracentral lobule; L, left; R, right*.

### Correlation Between Clinical Variables and DC in OSA Patients

To explore symptom-related whole-brain networks, we performed a correlation analysis between the DC values of brain areas that showed significant differences before and after treatment and the clinical data before and after treatment, which is shown in detail in Table 4.

**Table 4:** Correlation analyses between altered DC and clinical assessments in pre-and post-CPAP OSA patients.

**Brain areas**	**Clinical Assessments**	**r value**	***P* values**
**pre-CPAP OSA**
MTG.R	Min SaO2%	0.439	0.046
STG.L	HAMA	−0.647	0.002
PG/MFG.R	PSQI	−0.456	0.038
	HAMD	−0.515	0.017
MFG.L	ESS	−0.454	0.039
**post-CPAP OSA**
CPL	MoCA	0.453	0.039
MTG.R	MoCA	0.504	0.020
	PSQI	−0.454	0.039
STG.L	MoCA	0.48	0.028
PG.L	MoCA	0.467	0.033
PL.L	MoCA	0.512	0.018

*pre-CPAP, OSA patients before CPAP treatment; post-CPAP, OSA patients after CPAP treatment; HC, healthy controls; CPL, cerebellum posterior lobe; MTG, middle temporal gyrus; STG, superior temporal gyrus; PG, precentral gyrus; MFG, middle frontal gyrus; PL, paracentral lobule; L, left; R, right; SaO2, oxygen saturation; ESS, Epworth sleepiness scale; HAMA, Hamilton Anxiety Scale; HAMD, Hamilton Depression Scale; PSQI, Pittsburgh Sleep Quality Index; MoCA, Montreal Cognitive Assessment*.

Before CPAP treatment, the DC values of the right MTG in OSA were significantly correlated with the AHI and nadir SaO2. At the same time, the DC value of the left STG was significantly negatively correlated with HAMA scores, the DC value of the left PG and MFG was negatively correlated with PSQI and HAMD scores, and the DC value of the left MFG was negatively correlated with ESS scores. After one month of standard CPAP treatment, there was a significant positive correlation between the MoCA scores and the DC values of several brain regions, including the bilateral CPL, right MTG, left STG, and left PL. Moreover, we found a negative correlation between the PSQI scores and the DC value of the right MTG.

## Discussion

To our best knowledge, this study was the first to explore the changes in the intrinsic functional architecture of whole-brain functional networks in OSA patients before and after CPAP treatment using a DC approach based on whole-brain voxel levels. We found brain functional network reversal after CPAP treatment and that the brain areas reversed highly overlapped with the areas of initial damage, suggesting that the treatment is effective; these findings also provide an objective basis for clinical evaluation and potential mechanisms.

This study showed remarkably similar higher DC values in the specific brain region in both the OSA and NC groups, which generally overlaps with previous network hub studies (37). We also observed changes in network centrality in the bilateral CPL and right STG, left SFG, and right MFG, which were involved in cognitive and emotional processing. We further explored the changes in functional networks and clinical symptoms of OSA patients after CPAP treatment. The patients' drowsiness, anxiety and depression symptoms were relieved to varying degrees after CPAP treatment. This suggests that our CPAP treatment can improve the mood abnormalities of patients to some extent. Unexpectedly, compared with the baseline level itself, patients' sleep quality decreased after treatment, which we speculated was due to the patients' unfamiliarity and discomfort with the ventilator at the initial stage of wearing the ventilator. In addition, the increase in cognitive scores was not statistically significant after correction. Longer and more thorough treatment may be needed to achieve more significant improvements in cognitive function.

One month after CPAP treatment, the OSA patient group had significantly lower DC in the bilateral CPL with elevated DCs compared to the HC group and higher DCs in the frontal, temporal, and insular lobes, suggesting that CPAP can effectively reverse the functional network damage or compensatory response arising from OSA. Moreover, the decrease or increase in DC values in all brain regions of OSA patients after treatment was lower or higher than the level of DC values in the corresponding brain regions of the HC group, which may be the result of functional storage in OSA patients after short-term CPAP treatment. In addition, in the comparison before and after CPAP treatment, brain areas with reduced or elevated DC values after CPAP treatment overlapped highly with those in the baseline OSA patients with elevated or reduced DC values compared to the HC group, indicating that these brain areas are critical nodes that are vulnerable to injury in OSA patients and neuroreversible after CPAP treatment.

In the present study, the patients in the pre-CPAP OSA group showed increased DC values in the posterior cerebellar lobes compared to those of HCs. It is well-known that the cerebellum is associated with the regulation of respiratory patterns (38). Recent META analysis indicated enhanced cerebellar functional responses and reduced GMV in OSA patients compared to HCs (39), suggesting the possible existence of structurally vulnerable and functionally compensated areas of the cerebellum associated with OSA. This is consistent with our previous study finding increased intrinsic connectivity in the right posterior cerebellar lobe in OSA patients compared to HCs (10), which may be compensatory to adapt to exposure to long-term hypoxia conditions. The increased DC values in the bilateral CPL in this study may reflect increased connectivity within the CPL and presumably as an adaptive compensatory response to factors such as intermittent hypoxemia, hypercapnia, and sleep fragmentation in OSA patients. A longitudinal SPECT study found that CPAP treatment reversed reduced rCBF in the right CPL in OSA, and it proposed that the decreased cerebellar rCBF may be closely associated with the intermittent hypoxemia and sleep disturbances in OSA and could be reversed by standardized CPAP treatment (40). Some scholars have also found that long-term CPAP treatment can lead to an increase in the originally diminished cerebellar volume, which is thought to be due to the process of secondary compensatory neurology that occurs as a result of diminished oxidative stress in the neural tissue within a hypoxic environment (22). As we expected, after one month of CPAP treatment, the DC values in the bilateral CPL were reversed to a level lower than those of HC, suggesting that effective CPAP treatment can reverse cerebellar damage. We also found that after CPAP treatment, reduced DC values in the bilateral CPL in OSA were positively correlated with increased MoCA scores in OSA, suggesting that it may be involved in cognitive function repair. The cerebellum was susceptible to sleep fragmentation and hypoxia during sleep (14). Thus, we speculate that effective CPAP treatment can ameliorate the oxidative stress response caused by the patient's hypoxemia and disturbed sleep architecture, resulting in the reversal of cerebellar network integration and cerebellar connectivity.

Our study also found Pre-CPAP OSA showed decreased DC values in the left SFG and right MFG, while the DC values of the bilateral MFG, PG, and left PL in OSA were elevated after treatment and were higher than those of the HC group. These brain regions are important components of the frontal lobe, and most of them belong to the lateral prefrontal cortex (41, 42). Studies in humans and animals have shown that frontal areas play a central role in cognition, while the prefrontal cortex was closely associated with aspects of emotional processing and executive function (43, 44). Previous studies have found not only multiple brain functional abnormalities in the frontal lobes of OSA patients (11, 45, 46) but also brain structural damage (47, 48), suggesting that the frontal lobe was a functionally and structurally vulnerable area in OSA patients. This is consistent with the model proposed by Dean, W, in which sleep fragmentation and blood oxygen abnormalities in OSA patients affect sleep-related recovery processes at night, which then induce cytochemical and structural damage to the central nervous system. This results in impaired prefrontal cortex function, which manifests itself in daytime cognitive and behavioral impairments (49). Canessa et al. reported that gray-matter volume in specific frontal and hippocampal brain regions of OSA patients can be reversed by 3 month CPAP treatment, and these changes are significantly correlated with improvement in cognitive neuropsychologic tests (21). Consistent with previous studies, frontal network connectivity was improved after CPAP treatment, which may attributed to that effective CPAP treatment improves patients' sleep architecture and blood oxygen levels, allowing for efficient nocturnal frontal cortex functional recovery. After treatment, increased DC values in the PG and PL of OSA patients were positively correlated with posttreatment MoCA scores, which may probably involved in the pathology of OSA-related cognitive repairment.

The temporal lobe is closely related to the processing of memory and emotion (50, 51). Our previous study found reduced rCBF in the temporal lobe of OSA patients and reduced functional connectivity in prefrontal, parietal, and temporal brain regions in the DMN (12, 52), suggesting temporal lobe vulnerability to sleep fragmentation and hypoxemia injury. Consistent with the above findings, our study found that DC values in the right STG were reduced in pre-CAPA treatment of OSA and were positively correlated with nadir SaO2 and negatively correlated with HAMA. We speculated that this may be a functional connection disorder of the temporal cortex due to long-term exposure of the brain to ischemia and hypoxia in OSA patients, which may partially explain the mechanism of impaired emotion regulation in OSA patients. The DC values of the right MTG and left STG of patients increased after treatment and were higher than the levels in HCs group, which indicated that CPAP treatment could improve the intrinsic connectivity of patients with temporal lobe damage. This finding could provide new perspectives on the neuroimaging mechanisms of emotional processing in OSA patients treated with CPAP.

Previous studies have found reduced insula structure and function and metabolic abnormalities in OSA patients (53, 54), emphasizing the role of the insula in abnormal emotional and sensory processing in OSA patients (53). In our study, we found that DC values were elevated in the insula of patients after treatment. However, no significant difference in DC between the insula was found in baseline OSA patients compared with HCs. This may be due to the insula not being significantly damaged. In contrast, the insula of OSA patients, as a sleep fragmentation and chemic-hypoxic frontal structural and functional vulnerable area, was functionally enhanced after CPAP treatment.

### Limitations

There were several limitations of the study. First, only 21 treatment subjects were included for integration of global brain information and changes in clinical presentation, and these findings should be further validated with a larger sample. In addition, because no negative treatment group was established, we were unable to compare and observe whether OSA patients would show similar changes in brain function to our study after general supportive treatment. Finally, we only explored changes in brain function pivotal to one month of treatment and patients did not recheck PSG after treatment, and long-term treatment effects and its relationship with the restoration of sleep architecture need to be further explored.

## Conclusion

In conclusion, this was a novel study using voxelwise DC to investigate the functional brain network. We found a reversal of DC values in the brain network of OSA patients, mainly in the bilateral CPL, right MTG, left STG, right PG and MFG, left extranuclear and insula, left MFG, and left PL; these findings may indicate the neuroimaging mechanisms underlying the cognitive impairment in OSA patients, providing reliable imaging features and potential neuropathological mechanisms for clinical CPAP treatment, leading to the proposal of a new technique and direction for exploring the assessment of CPAP treatment in OSA patients.

## Data Availability Statement

The raw data supporting the conclusions of this article will be made available by the authors, without undue reservation.

## Ethics Statement

The studies involving human participants were reviewed and approved by the Medical Research Ethics Committee of The First Affiliated Hospital of Nanchang University [No. 2020(94)]. The patients/participants provided their written informed consent to participate in this study.

## Author Contributions

DP and HL guided and designs the MRI experiment. YS analyzed the resting-state fMRI data. PL and YS analyzed and discussed the ideas of the paper. PL unscrambled the results and wrote the manuscript. PL, XL, KL, LK, WX, and YZ collected resting fMRI data and applied for the ethics. HL and DP reviewed and revised the manuscript. All authors contributed to the article and approved the submitted version.

## Funding

This study was supported by the National Natural Science Foundation of China (Grant Nos. 81860307 and 81560285), the Natural Science Foundation Project of Jiangxi, China (Grant Nos. 20202BABL216036, 20181ACB20023, and 20171BAB205070), Education Department Project of Jiangxi Province, China (Grant Nos. 700544006 and GJJ190133), Department of Health Project and Jiangxi Province, China (Grant No. 20181039), and the Graduate Innovation Foundation of Jiangxi Province, China (Grant No. YC2020-S135).

## Conflict of Interest

The authors declare that the research was conducted in the absence of any commercial or financial relationships that could be construed as a potential conflict of interest.

## Publisher's Note

All claims expressed in this article are solely those of the authors and do not necessarily represent those of their affiliated organizations, or those of the publisher, the editors and the reviewers. Any product that may be evaluated in this article, or claim that may be made by its manufacturer, is not guaranteed or endorsed by the publisher.
