# Supplementary material for: The Effects of CPAP Treatment on Resting-State Network Centrality in Obstructive Sleep Apnea Patients
Source: Front Neurol. 2022 Mar 28;13:801121. doi: 10.3389/fneur.2022.801121 (PMC8995649; doi:10.3389/fneur.2022.801121)
Supplement: Supplementary file 1 [file Table_1.DOCX]

Supplementary Material

# Supplementary Figures
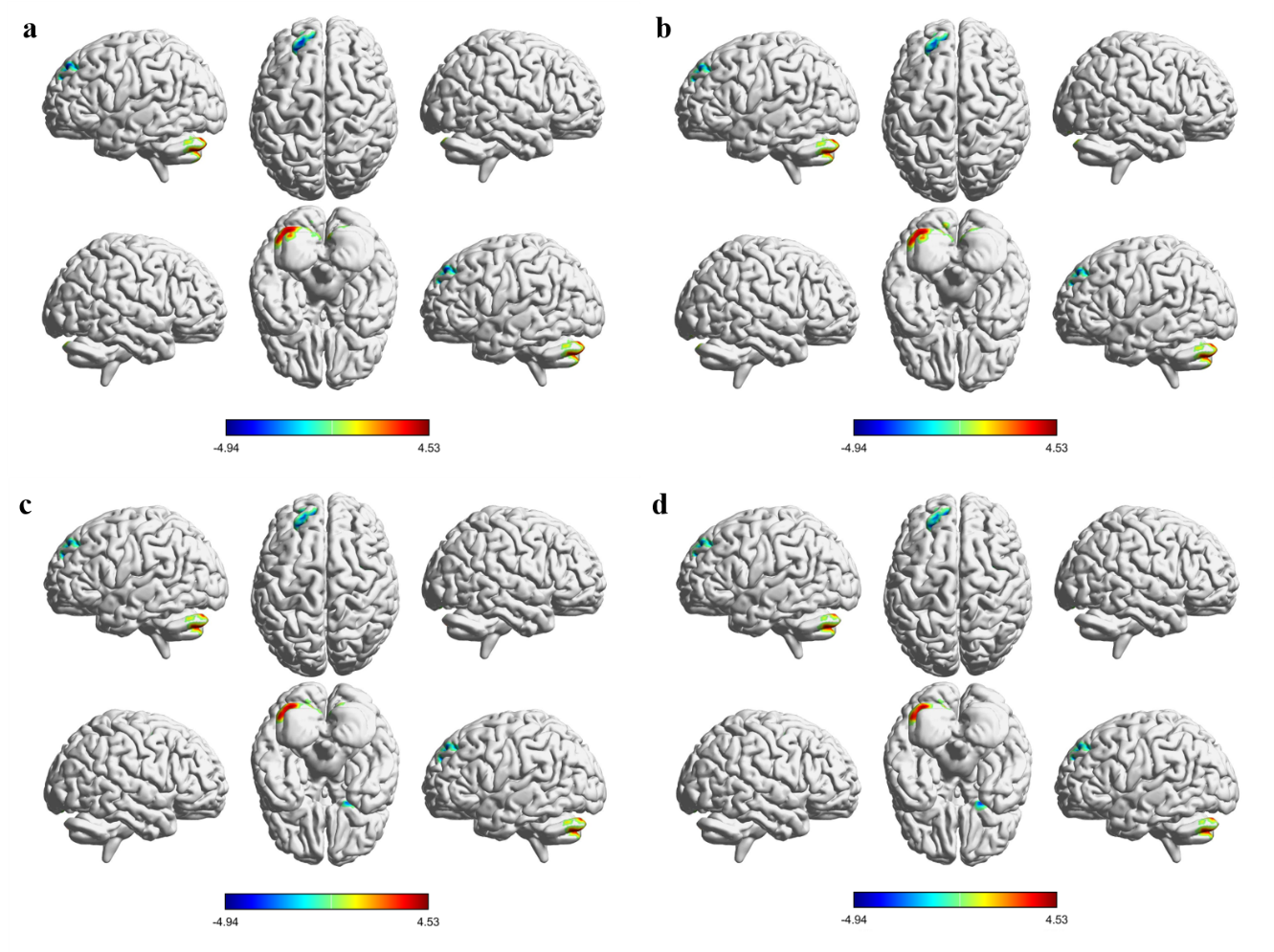
Supplementary Figure 1. Voxelwise comparison of DC between patients in the Pre-CPAP and HC groups at different correlation thresholds (r0= 0.15, 0.2, 0.3 and 0.35) (a~d).


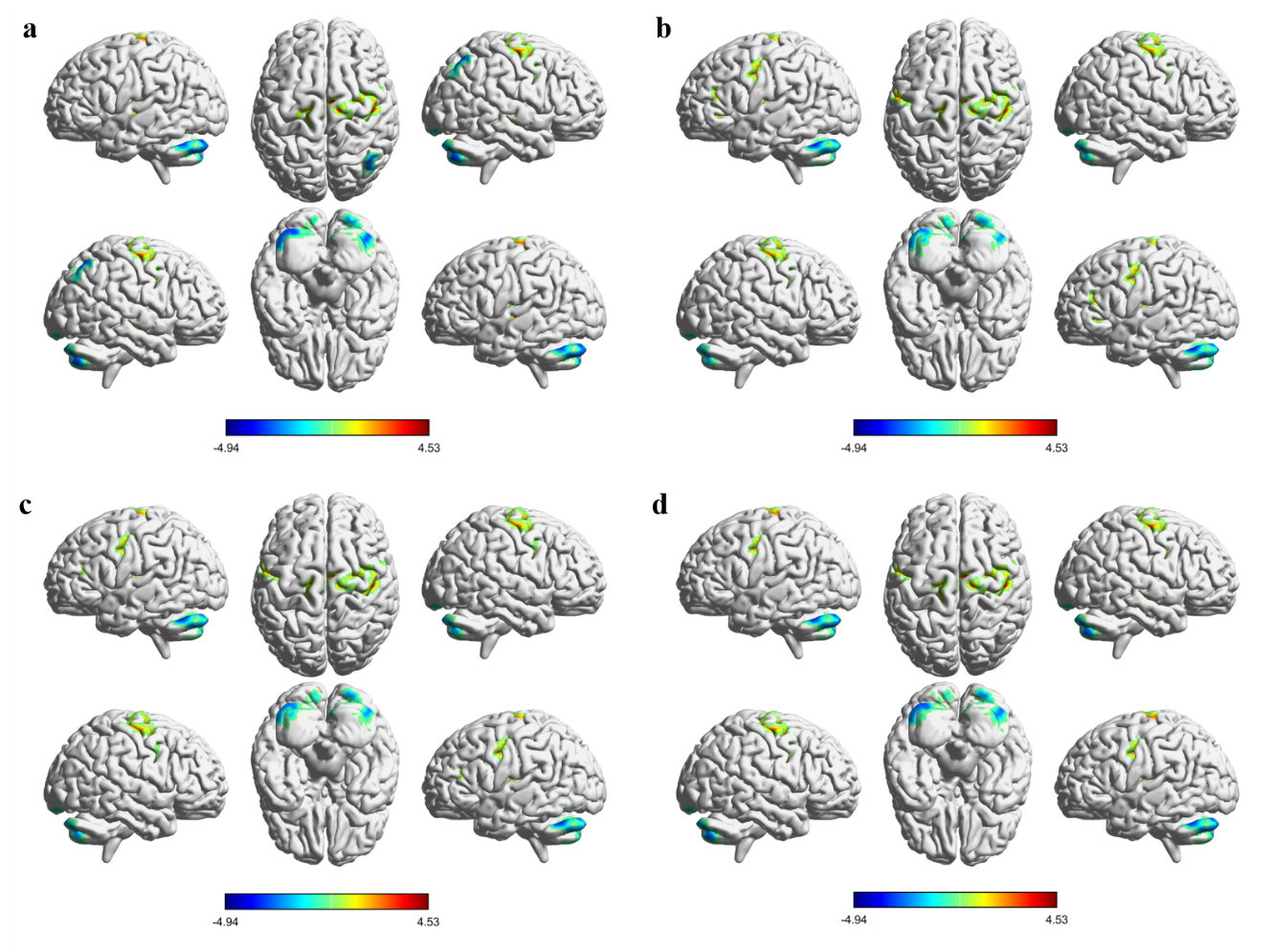


**Supplementary Figure 2.** Voxelwise comparison of DC between patients in the Post-CPAP and Pre-CPAP OSA groups at different correlation thresholds (r0= 0.15, 0.2, 0.3 and 0.35) (a~d).
